# Thrombopoietin is required for full phenotype expression in a *JAK2*^V617F^ transgenic mouse model of polycythemia vera

**DOI:** 10.1371/journal.pone.0232801

**Published:** 2020-06-01

**Authors:** Jerry L. Spivak, Akil Merchant, Donna M. Williams, Ophelia Rogers, Wanke Zhao, Amy Duffield, Linda S. Resar, Alison R. Moliterno, Zhizhuang J. Zhao

**Affiliations:** 1 Hematology Division, Department of Medicine, Johns Hopkins University School of Medicine, Baltimore, Maryland, United States of America; 2 Samuel Oschin Comprehensive Cancer Institute, Blood and Marrow Transplant Program, Cedars-Sinai Medical Center, Los Angeles, California, United States of America; 3 Department of Pathology, University of Oklahoma Health Sciences Center, Oklahoma City, Oklahoma, United States of America; 4 Department of Pathology, Hematologic Pathology, Johns Hopkins University School of Medicine, Baltimore, Maryland, United States of America; Wayne State University, UNITED STATES

## Abstract

The myeloproliferative neoplasms, polycythemia vera, essential thrombocytosis and primary myelofibrosis are hematopoietic stem cell disorders and share driver mutations that either directly activate the thrombopoietin receptor, MPL, or activate it indirectly through gain-of-function mutations in the gene for JAK*2*, its cognate tyrosine kinase. Paradoxically, MPL surface expression in hematopoietic stem cells is also reduced in the myeloproliferative neoplasms due to abnormal post-translational glycosylation and premature destruction of JAK2, suggesting that the myeloproliferative neoplasms are disorders of MPL processing since MPL is the only hematopoietic growth factor receptor in hematopoietic stem cells. To examine this possibility, we genetically manipulated MPL expression and maturation in a *JAK2*^V617F^ transgenic mouse model of polycythemia vera. Elimination of MPL expression completely abrogated the polycythemia vera phenotype in this *JAK2*^V617F^ transgenic mouse model, which could only be partially restored by expression of one *MPL* allele. Most importantly, elimination of thrombopoietin gene expression abrogated the polycythemia vera phenotype in this *JAK2*^V617F^ transgenic mouse model, which could be completely restored by expression of a single thrombopoietin allele. These data indicate that polycythemia vera is in part a thrombopoietin-dependent disorder and that targeting the MPL-thrombopoietin axis could be an effective, nonmyelotoxic therapeutic strategy in this disorder.

## Introduction

The myeloproliferative neoplasms (MPN), polycythemia vera (PV), essential thrombocytosis (ET) and primary myelofibrosis (PMF) are clonal hematopoietic stem cell (HSC) disorders that share gain of function mutations which directly or indirectly constitutively activate JAK2 [1–4], the cognate tyrosine kinase of the erythropoietin (EPO) and thrombopoietin (THPO) receptors [5], and also utilized by the granulocyte colony-stimulating factor receptor [6]. Constitutive JAK2 activation accounts for increased blood cell production in the MPN because JAK2 is responsible for the proliferation and survival of committed hematopoietic progenitor cells (HPC) [7,8]. Ruxolitinib, a JAK1/2 inhibitor, is effective in controlling unregulated MPN HPC proliferation [9]. The MPN, however, are HSC disorders and JAK2^V617F^ did not alter MPN HSC pool size nor did JAK2 inhibition significantly reduce the MPN HSC burden in animal [10] or human studies [11], suggesting mechanisms other than JAK2 activation are also involved in MPN pathophysiology.

HSC express only one hematopoietic growth factor receptor, the THPO receptor, MPL. JAK2 is the obligatory chaperone for MPL cell-surface expression and stability [12]. THPO promotes HSC survival [13] and megakaryocytic progenitor cell proliferation [14] but is not required for megakaryocyte maturation or platelet production [15,16]. Its major role in adult hematopoiesis is maintenance of HSC quiescence within the bone marrow osteoblastic niche [17,18]. Adult mice lacking the *MPL* or *THPO* gene, appear normal except for thrombocytopenia but have a marked increase in plasma THPO and a decrease in marrow HSC [19]. In humans with congenital amegakaryocytic thrombocytopenia (CAMT), *MPL* loss of function mutations, usually in the MPL distal extracellular cytokine receptor homology domain (CRHD) [20], cause thrombocytopenia, an elevated plasma THPO level, and progressive marrow aplasia [21].

With respect to the MPN, *MPL* is a proto-oncogene since the retrovirus MPLV, which encodes an *MPL* gene truncated in its extracellular domain, caused an acute, fatal PV-like syndrome in mice [22], and in vitro, immortalized murine HPC [23]. Ectopic THPO-producing murine bone marrow cells caused a fatal transplantable myeloproliferative disorder with splenomegaly, osteomyelofibrosis, pancytopenia and leukemic transformation [24,25]. In contrast, ectopic EPO expression in murine marrow cells [26] or erythroid progenitor cell-specific expression of *JAK2*^V617F^ caused erythrocytosis without significant extramedullary hematopoiesis (EMH) and failed to propagate the erythrocytosis phenotype in secondary recipients [10], emphasizing the primary role of HSC, MPL and THPO in MPN pathophysiology.

In humans, hereditary or acquired *MPL* mutations involving the transmembrane domain or distal CRHD are associated with an ET or PMF phenotype [2,27,28]. Furthermore, mutated *CALR* binds and activates MPL causing an ET or PMF phenotype [29–31]. Importantly, germline single nucleotide polymorphisms (SNP) involving the *MPL* distal CRHD were associated with a variably penetrant, benign thrombocytosis phenotype with an elevated plasma THPO level, were ethnic group-specific [32, 33] and could be modeled in the mouse [34, 35]. Hereditary *THPO* mutations permitting unregulated THPO production caused thrombocytosis alone [36], but in one family were associated with leukemic transformation or myelofibrosis [37].

In contrast to *MPL* mutations, *JAK2*^V617F^ causes PV, ET and PMF. However, like hereditary or acquired *MPL* [2,38] and *CALR* mutations [29], impaired MPL cell-surface expression is a feature of *JAK2*^V617F^-positive PV, ET and PMF [34–42] and presumably responsible for increased plasma THPO in these disorders [34,43,44]. But how impaired expression of the hematopoietic growth factor receptor responsible for HSC maintenance, expansion and thrombopoiesis could cause myeloproliferation has been a conundrum. To examine this issue, we genetically manipulated *MPL* and *THPO* expression in a *JAK2*^V617F^ transgenic mouse model of PV [45]. Our results indicate that despite expression of constitutively-active JAK2^V617F^, the PV phenotype in this mouse model still required THPO signaling and suggest that interfering with the MPL-THPO interaction could have therapeutic value treating the MPN.

## Materials and methods

### Generation of murine models

This research project involved the use of mice and was approved by the Johns Hopkins University School of Medicine IACUC under protocol number M013M467. Isofluorane was used for anesthesia and CO2 narcosis and cervical dislocation was used for euthanasia.

Generation of transgenic mice expressing 13 copies of the entire coding region of human *JAK2*^V617F^ plus the 3’ noncoding region cloned into the HS321/45-vav vector and crossed into a C57Bl/6 background has previously been described [45]. Wild-type C57BL/6 mice were obtained from the Jackson Laboratory (Bar Harbor, ME). *MPL* knockout mice [46] and *THPO* knockout mice [15] created by homologous recombination using a targeting vector containing a neomycin-resistance (neo^r^) cassette in C57Bl/6 mice were obtained from Genentech. Experiments were performed using mice 6–9 weeks of age or older. Mice were raised in approved housing and all experimental protocols were approved by our institutional ACUC (Protocol #M013M467).

### Genotyping

Mice were genotyped using tail snips obtained at 3–5 weeks of age. The genotyping primers are shown in the S1 Table in the Supplemental information.

### Hematological analysis

Mice were anesthetized with isofluorane (Baxter NDC 10019-360-60) and tail vein blood (100 μL) was collected in K-EDTA. Complete blood and differential counts were performed with a Hemavet 950FS (Drew Scientific) using the manufacturer’s mouse program and controls (Mouse Multi-trol 600065).

### Histopathology

Mice were sacrificed and the spleen and femurs were removed and placed in formalin, paraffin-embedded and stained with hematoxylin and eosin for morphology, and silver-stained for analysis of reticulin formation.

### Thrombopoietin assay

The plasma thrombopoietin concentration was measured using an ELISA assay ((Quantikine ELISA; catalogue # MTP00, R&D Systems, Minneapolis, MN) according to the manufacturer’s specifications.

### Hematopoietic progenitor cell (HPC) colony-forming analysis

CFU-GEMM, CFU-GM and BFU-E colony formation was assessed in vitro using washed suspensions of 5 X10^4^ marrow cells or 3 X 10^5^ spleen cells suspended in IMDM and plated in 1% methylcellulose with 30% FBS (Methocult medium, catalogue #3534, STEMCELL Technologies, Vancouver, BC Canada) containing 10 ng/μL of mIL3 (catalogue #02733, STEMCELL), 10 ng/μL of rhIL6 (#206-IL-010, R&D SYSTEMS, Minneapolis, MNUSA), 50ng/μL of THPO (#288-TP-005, R&D) and 3 U/μL of rhEPO (Johnson and Johnson, New Brunswick, NJ USA). All colony-forming assays were performed between weeks 15–18. Colony-forming assays were performed in triplicate with at least 3 replicate experiments and colony formation was assessed at 7 days. CFU-Mk colony formation was assessed using 5 X10^4^ washed marrow cells or 3 X 10^5^ spleen cells suspended in IMDM and plated in methylcellulose (Megacult-C medium, # 04850, STEMCELL) containing 10 ng/μL of mIL3, 10 ng/μL of rhIL6 and 50ng/μL of THPO, R&D). Colony number was assessed at 7 days after staining with acetylcholinesterase activity as described in the Megacult-C protocol for murine cells.

### Bone marrow flow cytometry

Bone marrow from three to four mice per experimental group was flushed from the femurs and tibias with staining medium (RPMI with 2% FBS), filtered and suspended at a concentration of 10^8^ cells/mL in staining medium. The antibodies used for staining were: CD34 (Clone: RAM34)-allophycocyanin (APC) or FITC; FcRγ (Clone: 93)-PE or Flt3 (clone: A2F10)-PE; c-Kit (Clone: 2B8)-APC-Alexa Fluor 750; Sca1 (Clone: D7)–PE-cyanin 7; CD150 (Clone: TC15-12F12.2)-APC (BioLegend, San Diego, CA USA); CD48 (Clone: HM48.1)-PE (BD Biosciences, San Jose, CA USA) and biotin-streptavidin-peridinin-chlorophyll-protein complex-cyanin 5.5 (PerCP-Cy5.5) or eFluor450-labeled lineage cocktail (CD3e, Gr1, B220 and Ter119) (all from Thermo Fisher Scientific, Waltham, MA USA). Apoptotic cells were identified with the FITC Annexin V Apoptosis Kit (BD Biosciences). Labeled cells were analyzed on a 9 laser LSRII (BD Biosciences) [47].

### Statistical analysis

Pairwise individual significance was determined using either Student’s T-test or, if the normality or equal variance test failed, the Mann-Whitney Rank Sum test. In addition, an all pairwise multiple comparison procedure (Dunn’s Method) was performed on each group of age-matched hemoglobin, neutrophil and platelet levels from the four genotypes resulting from each cross (e.g. wild-type, *JAK2*^V617F^, *MPL*^del/del^, *MPL*^del/+^, *JAK2*^V617F^*/MPL*^del/del^, *JAK2*^V617F^/*MPL*^del/+^). Calculations were performed using Sigma Plot (Systat Software, San Jose, CA).

## Results

### Breeding strategy

To study the role of MPL in the PV phenotype, we used a *JAK2*^V617F^ transgenic mouse, which develops erythrocytosis, leukocytosis and thrombocytosis with an eventual decline in erythropoiesis associated with EMH and osteomyelofibrosis over a time course of 6–46 weeks without leukemic transformation [45]. To manipulate MPL expression, we bred *JAK2*^V617F^ transgenic mice with *MPL*^del/del^ mice to obtain *JAK2*^V617F^ transgenic mice either null or heterozygous for the *MPL* gene. The breeding strategy (S1 Fig) yielded the expected Mendelian allelic ratios with the exception of slightly fewer than expected *JAK2*^V617F^/*MPL*^del/del^ mice. However, all mice were robust, had weights compatible with their sex and there was no excess mortality or sex-related differences with respect to blood counts in any genotype (S2 Table).

### Effect of the *JAK2*^V617F^ transgene on the blood counts, marrow and spleen HPC colony formation, marrow and spleen histology, spleen weights and the plasma THPO level

**Fig 1:**
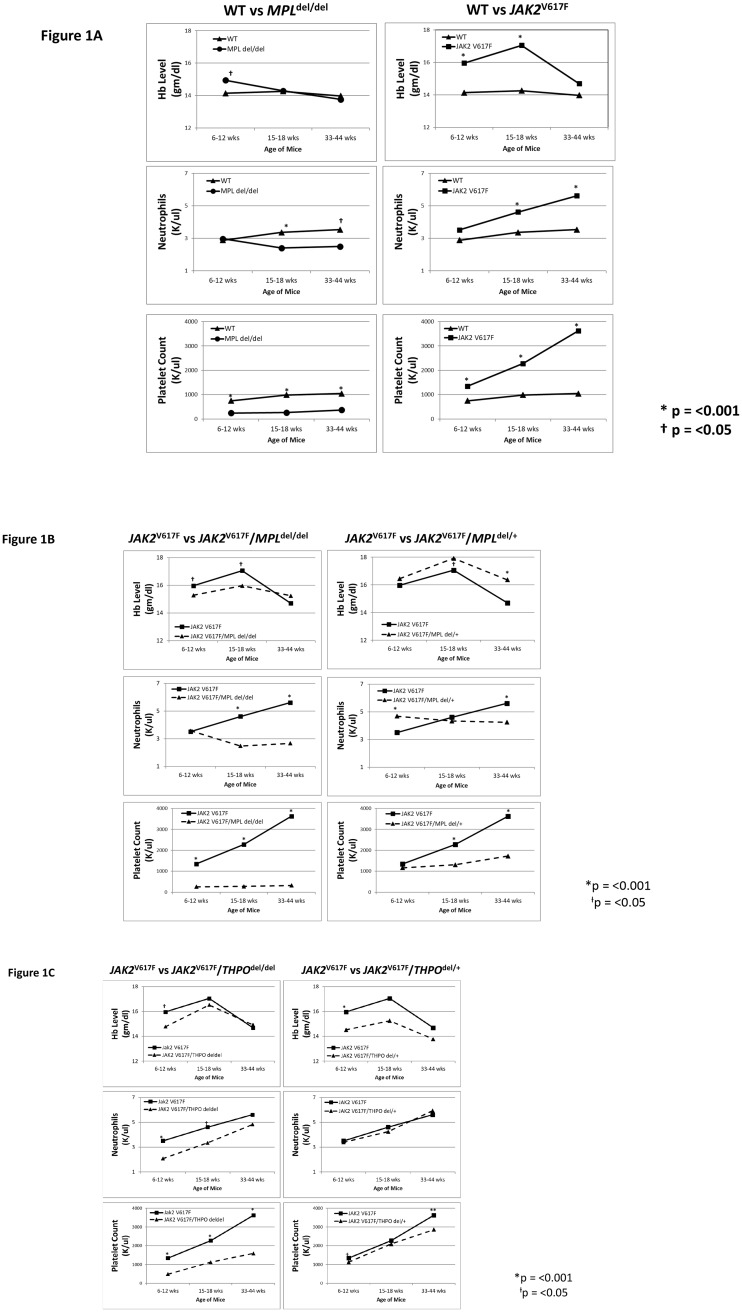
*MPL*^*del*^ and *THPO*^*del*^ genotypes mitigate increased hemoglobin, neutrophil and platelet levels in a *JAK2*^V617F^ transgenic mouse model of PV. (A) Age-matched hemoglobin, neutrophil and platelet levels in wild-type (WT) mice compared to *MPL*^del/del^ and *JAK2*^*V617F*^ transgenic mice over a period of 6 to 46 weeks. The symbols indicate statistically significant differences. The number of mice studied and complete statistical analysis for all genetic crosses is in the S3 Table in the Supporting information. (B) Age-matched hemoglobin, neutrophil and platelet levels in *JAK2*^*V617F*^ transgenic mice compared to *JAK2*^*V617F*^*/MPL*^*del/del*^ and *JAK2*^*V617F*^*/MPL*^*del/+*^ transgenic mice. (C) Age-matched hemoglobin, neutrophil and platelet levels in *JAK2*^*V617F*^ transgenic mice compared to *JAK2*^*V617F*^*/*THPO^del*/del*^ and *JAK2*^*V617F*^*/THPO*^*del/+*^ transgenic mice. *P < 0.001; ^Ɨ^P <0.05.

**Fig 2:**
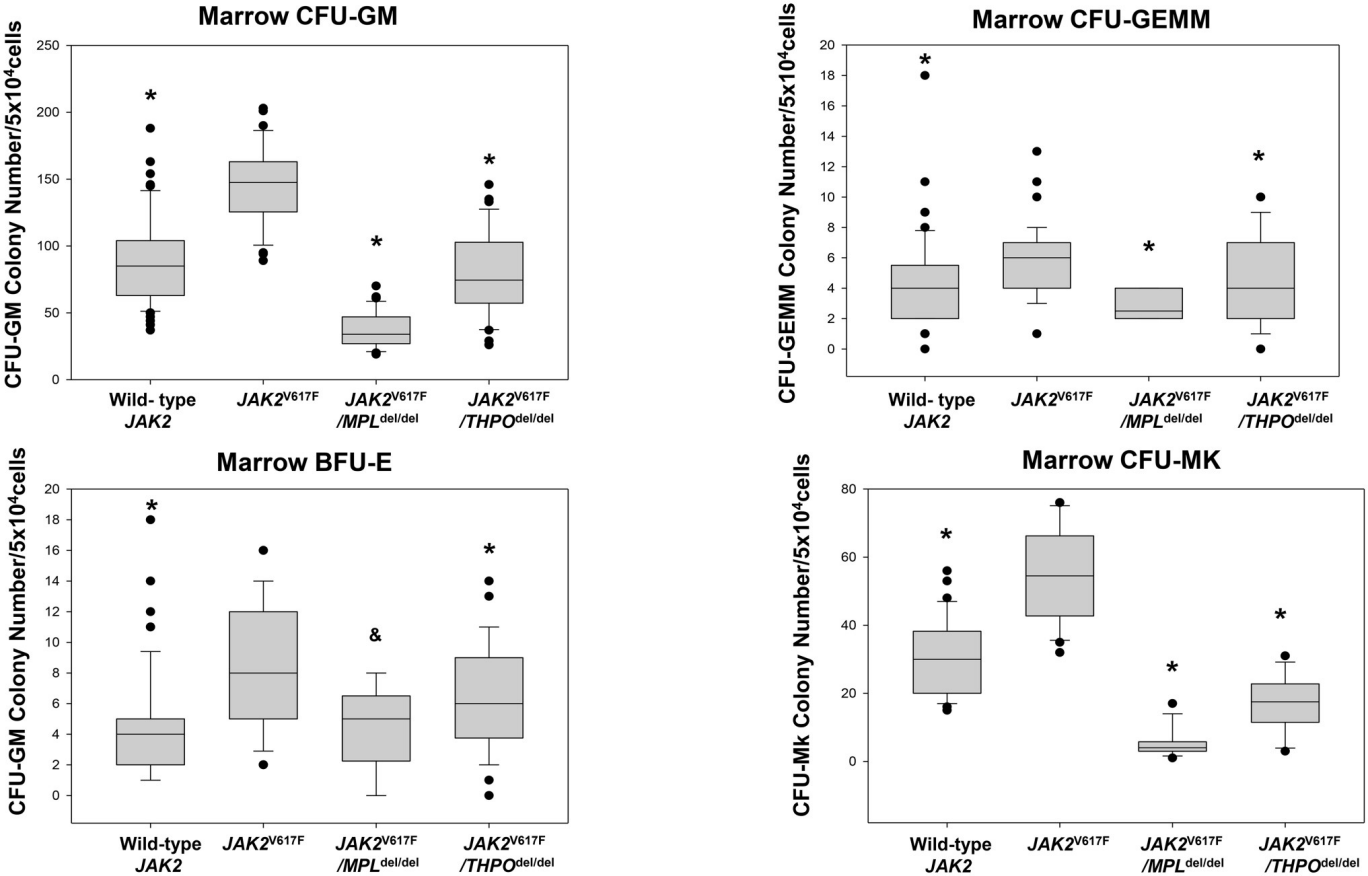
*MPL*^*del*^ and *THPO*^*del*^ genotypes mitigate marrow HPC colony formation in a *JAK2*^V617F^ transgenic mouse model of PV. (A) In vitro colony formation by marrow CFU-GM from *JAK2*^V617F^ transgenic mice (n = 10) was increased compared to wild-type mice (n = 10) (*P < 0.001), but was reduced below the wild-type level in *JAK2*^*V617F*^*/MPL*^*del/del*^ (n = 6) transgenic mice (*P < 0.001). By contrast, CFU-GM colony formation by *JAK2*^*V617F*^*/THPO*^*del/del*^ transgenic mice (n = 6) was similar to wild-type mice. (B) In vitro colony formation by marrow CFU-GEMM from *JAK2*^V617F^ transgenic mice was increased compared to wild-type mice and *JAK2*^*V617F*^*/MPL*^*del/del*^ and *JAK2*^*V617F*^*/THPO*^*del/del*^ transgenic mice (*P < 0.001). (C) In vitro colony formation by marrow BFU-E from *JAK2*^V617F^ transgenic mice was increased compared to wild-type mice and *JAK2*^*V617F*^*/MPL*^*del/del*^ and *JAK2*^*V617F*^*/THPO*^*del/del*^ transgenic mice (*P < 0.001). (D) In vitro colony formation by marrow CFU-Mk from *JAK2*^V617F^ transgenic mice was increased compared to wild-type mice and *JAK2*^*V617F*^*/MPL*^*del/del*^ and *JAK2*^*V617F*^*/THPO*^*del/del*^ transgenic mice (*P < 0.001). Importantly, HPC colony formation also provides insight into the contribution of JAK2^*V617F*^ compared to THPO in this transgenic mouse model based on HPC colony formation in the absence of THPO. This was best observed with CFU-GM and CFU-Mk, for which the increments induced by JAK2^*V617F*^ were the most robust: for CFU-GM, the increment induced by JAK2^*V617F*^ was abolished by the absence of THPO, while for CFU-Mk, colony formation by JAK2^*V617F*^ in the absence of THPO was 50% of wild-type. The horizontal lines of the boxes indicate the 25^th^ percentile, the median and the 75^th^ percentile respectively and the error bars indicate the 10^th^ and 90^th^ percentiles. *P <0.001; ^§^P<0.02. The number (n) of mice of each genotype used in all the experiments is in parentheses.

**Fig 3:**
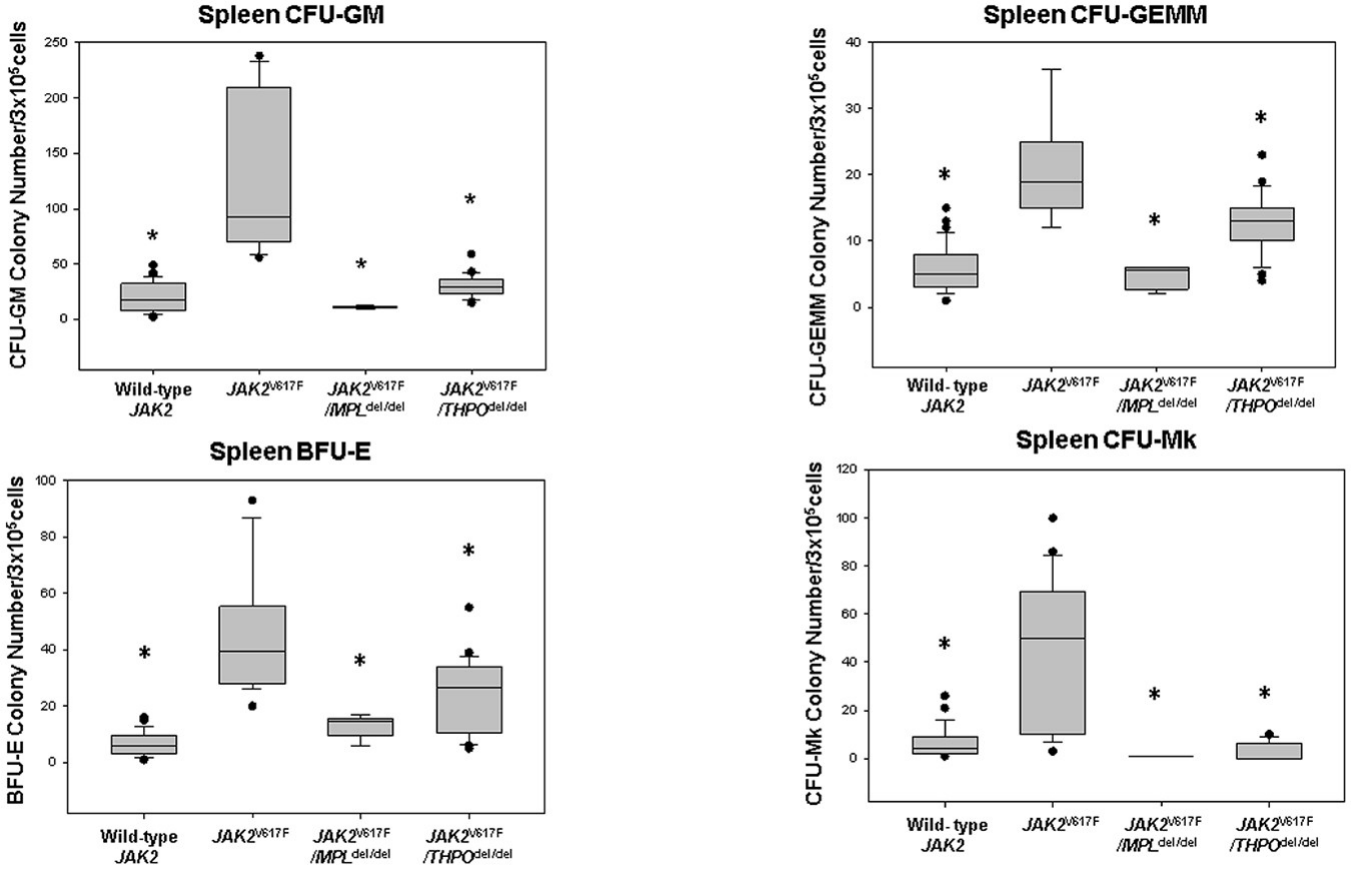
*MPL*^del^, and *THPO*^del^ genotypes mitigate spleen HPC colony formation in a *JAK2*^V617F^ transgenic mouse model of PV. (A) In vitro colony formation by spleen CFU-GM from *JAK2*^V617F^ transgenic mice was increased compared to wild-type mice and *JAK2*^V617F^*/MPL*^del/del^ and *JAK2*^V617F^*/THPO*^del/del^ transgenic mice (*P < 0.001). (B) In vitro colony formation by spleen CFU-GEMM from *JAK2*^V617F^ transgenic mice was increased compared to wild-type mice and *JAK2*^V617F^*/MPL*^del/del^ and *JAK2*^V617F^*/THPO*^del/del^ transgenic mice (*P < 0.001). (C) In vitro colony formation by spleen BFU-E from *JAK2*^V617F^ transgenic mice was increased compared wild-type mice and *JAK2*^V617F^*/MPL*^del/del^ and *JAK2*^V617F^*/THPO*^del/del^ transgenic mice. (*P < 0.001) (D) In vitro colony formation by spleen CFU-Mk from *JAK2*^V617F^ transgenic mice was increased compared to wild-type mice and *JAK2*^V617F^*/MPL*^del/del^ and *JAK2*^V617F^*/THPO*^del/del^ transgenic mice (*P < 0.001). The horizontal lines of the boxes indicate the 25^th^ percentile, the median and 75^th^ percentile respectively and the error bars indicate the 10^th^ and 90^th^ percentiles.*P <0.001. The number of mice of each genotype used in all the experiments is the same as in Fig 2.

**Fig 4:**
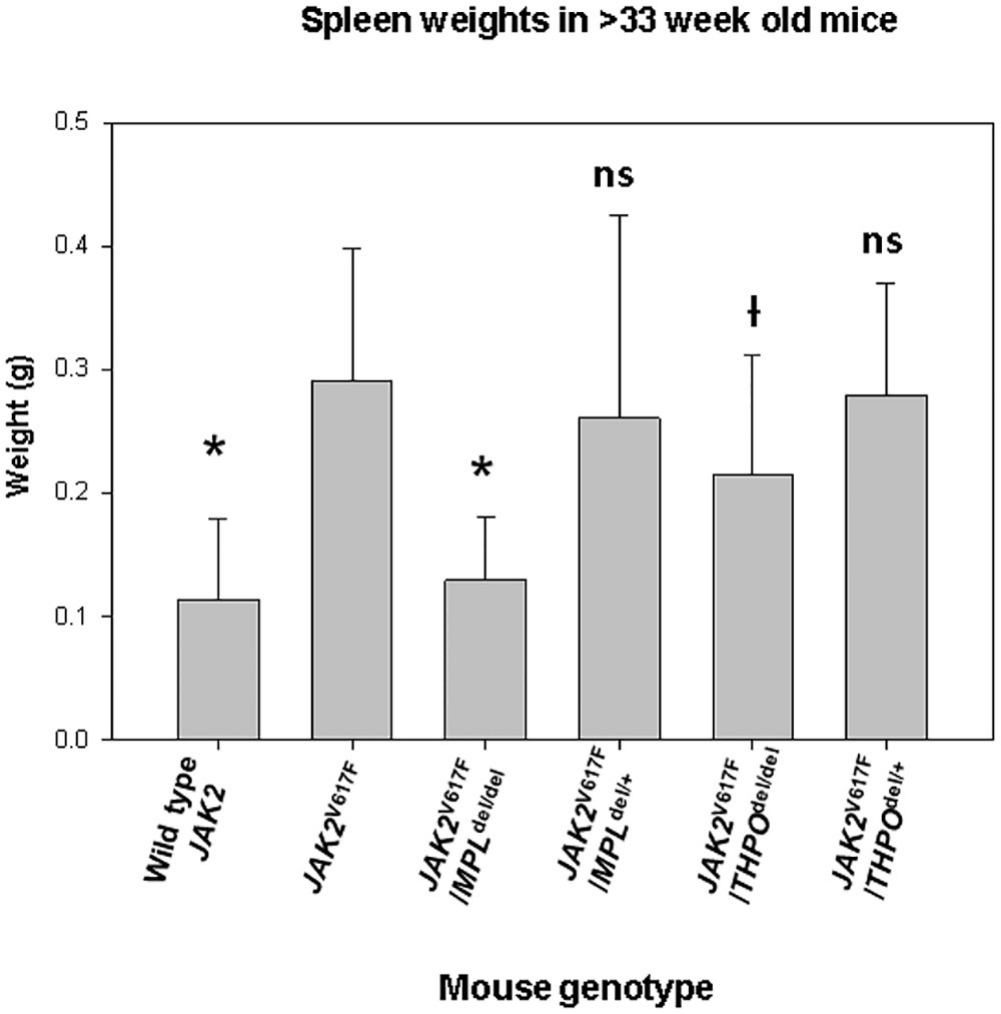
*MPL*^del^ and *THPO*^del/del^ genotypes mitigate splenomegaly in a *JAK2*^V617F^ transgenic mouse model of PV while splenomegaly was restored *JAK2*^V617F^*/THPO*^del/+^ mice. Spleen weights at > 33 weeks in wild-type mice (n = 39) and *JAK2*^V617^ (n = 53), *JAK2*^V617F^*/MPL*^del/del^ (n = 20), *JAK2*^V617F^*/MPL*^del/+^ (n = 22), *JAK2*^V617F^*/THPO*^del/del^ (n = 20) and *JAK2*^V617F^*/THPO*^del/+^ (n = 34) transgenic mice. The data are shown as means +/- the standard deviation. *P <0.001; ^Ɨ^P <0.004; ns = not significant compared to *JAK2*^V617F^. The number (n) of mice of each genotype studied is in parentheses.

**Fig 5:**
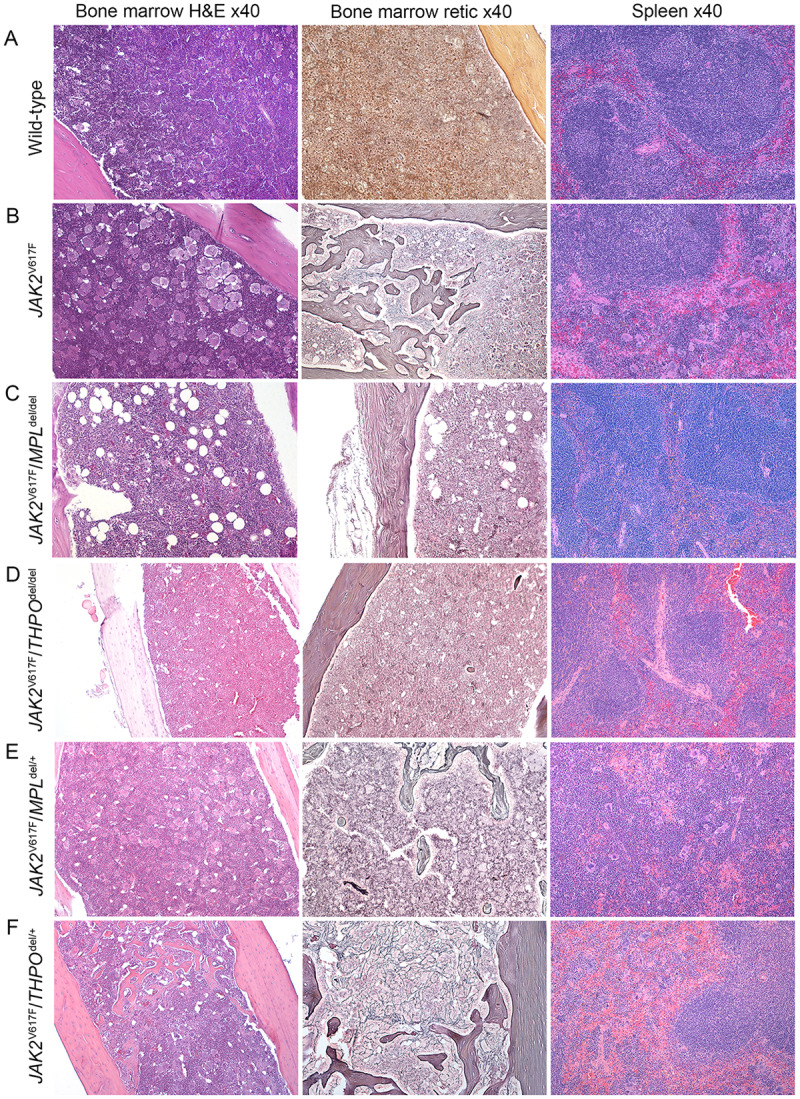
*MPL*^del^ and *THPO*^del/del^ genotypes mitigate marrow and spleen histopathology in a *JAK2*^V617F^ transgenic mouse model of PV but this was restored in *JAK2*^V617F^*/THPO*^del/+^ mice. Representative marrow and spleen histology at > 33 weeks in wild-type mice and *JAK2*^V617F^, *JAK2*^V617F^*/MPL*^del/del^ *JAK2*^V617F^*/THPO*^del/del^, *JAK2*^V617F^*/MPL*^del/+^, and *JAK2*^V617F^*/THPO*^del/+^ transgenic mice. All images were taken with Zeiss AX10 Imager microscope using a Plano-APO 10X, 0.45 NA lens with a tungsten 3200 Kelvin light source. The imaging medium was digital photomicrography using bright field microscopy and a Pro Res 14 camera with Adobe Photoshop CC acquisition software. Magnification was 40X for all images.

**Fig 6:**
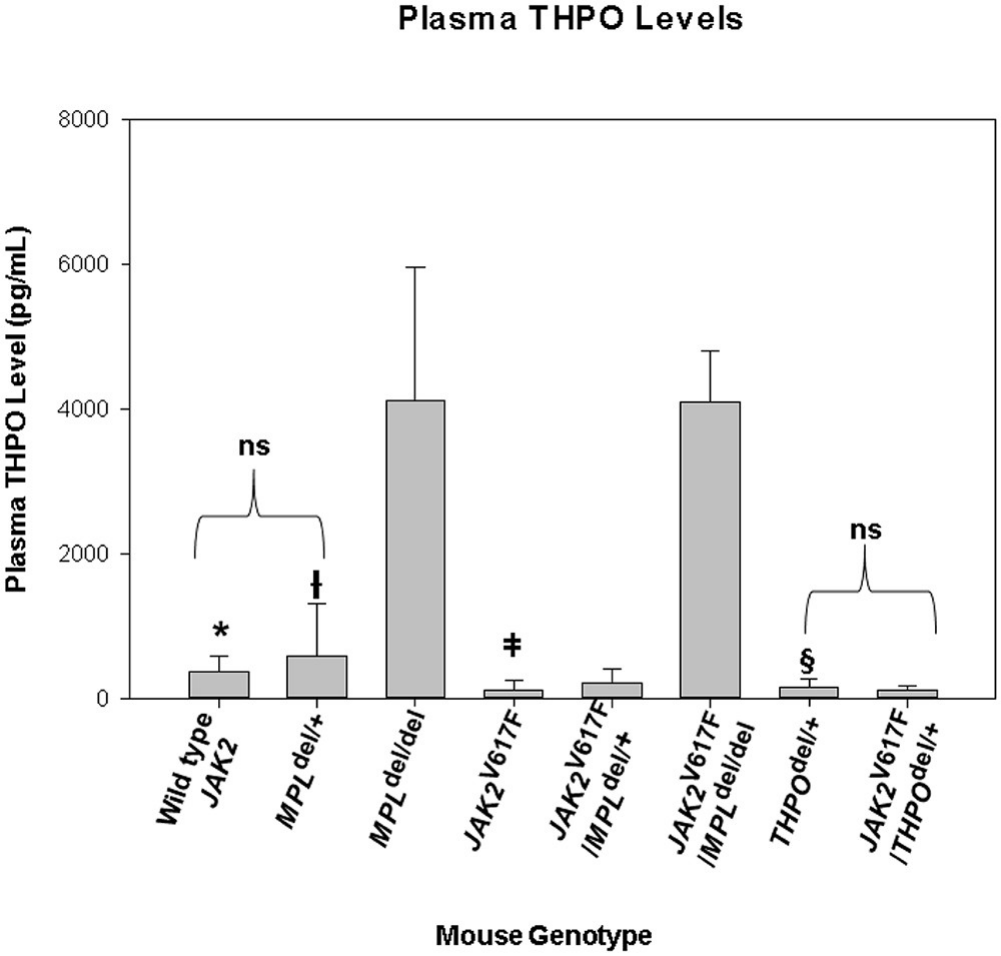
*MPL*^del^ and *THPO*^del^ genotypes alter the plasma THPO level in a *JAK2*^V617F^ transgenic mouse model of PV. Plasma THPO was measured in wild-type (WT) (n = 26), *MPL*^del/+^ (n = 16), *MPL*^del/del^ (n = 8) and *THPO*^del/+^ (n = 11) mice and *JAK2*^V617F^ (n = 21), *JAK2*^V617F^*/MPL*^del/+^ (n = 13), *JAK2*^V617F^*/MPL*^del/del^ (n = 8) and *JAK2*^V617F^*/THPO*^del/+^ (n = 4) transgenic mice. WT vs *JAK2*^V617F^, *P <0.001; *MPL*^del/+^ vs *JAK2*^V617F^*/MPL*^del/+^, ^Ɨ^P < 0.01; *JAK2*^V617F^ vs *JAK2*^V617F^*/MPL*^del/+^, ^ǂ^P = 0.004; WT vs *THPO*^del/+^, ^§^P <0.001. The data are shown as means +/- the standard deviation. ns = not significant. The number (n) of mice of each genotype studied is in parentheses.

There were slight but consistent differences in the hemoglobin level and neutrophil counts in *MPL*^del/del^ mice as well as the expected marked difference in their platelet counts. In *JAK2*^V617F^ transgenic mice, the hemoglobin level was initially higher than in the wild-type mice, rose further by 14–16 weeks and fell to the level of wild-type mice by 33 weeks (Fig 1A). The neutrophil count was initially normal, a feature seen in many PV patients, but increased thereafter, while the platelet count was elevated at 6–9 weeks and progressively increased. HPC colony-forming assays at 16 weeks revealed that *JAK2*^V617^ enhanced the number of marrow (Fig 2A–2D) and spleen (Fig 3A–3D) CFU-GEMM, CFU-GM, BFU-E and CFU-Mk compared to wild-type mice. Splenomegaly developed after 33 weeks of age (Fig 4), along with osteomyelofibrosis and splenic EMH (Fig 5), while plasma THPO was reduced relative to the wild-type mouse, indicating increased THPO utilization (Fig 6).

### Loss of MPL expression mitigated the PV phenotype of the *JAK2*^V617F^ transgenic mouse

*MPL* gene elimination in the *JAK2*^V617F^ transgenic mouse (*JAK2*^V617F^/*MPL*^del/del^) reduced the platelet count to the level of *MPL*^del/del^ mice, prevented an increase in the neutrophil count, and reduced but did not completely normalize the hemoglobin level (Fig 1B). *JAK2*^V617F^/*MPL*^del/del^ mice had reduced numbers of marrow (Fig 2A–2D) and spleen (Fig 3A–3D) CFU-GEMM, CFU-GM, BFU-E and CFU-Mk. Spleen weight was also reduced in these mice after 33 weeks (Fig 4) and, most importantly, there was complete reversal of the osteomyelofibrosis and splenic EMH associated with JAK2^V617F^ expression (Fig 5), while plasma THPO was markedly elevated (Fig 6).

Expression of one *MPL* allele (*JAK2*^V617F^/*MPL*^del/+^) restored erythrocytosis but not the neutrophilic leukocytosis or thrombocytosis (Fig 1B). Marrow and spleen histology (Fig 5) associated with the *JAK2*^V617F^ phenotype was partially restored, as was spleen size at 33 weeks (Fig 4). The plasma THPO level was markedly reduced compared to the *JAK2*^V617F^/*MPL*^del/del^ mouse but was still higher than in the *JAK2*^V617F^ transgenic mouse (Fig 6), supporting increased THPO utilization with *JAK2*^V617F^ expression.

### Loss of THPO expression abrogated the PV phenotype in the *JAK2*^V617F^ transgenic mouse

Since plasma THPO was not elevated in the *JAK2*^V617F^ transgenic mouse compared to wild-type mouse, suggesting that THPO contributed to the PV phenotype, we bred *JAK2*^V617F^ mice with *THPO*^del/del^ mice. In the absence of THPO in *JAK2*^V617F^/*THPO*^del/del^ mice, erythrocytosis was partially suppressed at 6–12 weeks, the neutrophil count was suppressed until after 15–18 weeks, and the platelet count was reduced to normal at all-time points (Fig 1C); marrow (Fig 2A–2D) and spleen (Fig 3A–3D) CFU-GEMM, CFU-GM, and CFU-Mk numbers were reduced but not marrow BFU-E, there was a reduction in spleen weight, though not to normal, (Fig 4) and, most strikingly, reversal of the osteomyelofibrosis and splenic EMH (Fig 5).

In contrast to restoration of one *MPL* allele in the *JAK2*^V617F^/*MPL*^del/+^ mouse (Fig 1B), restoration of one *THPO* allele in the *JAK2*^V617F^ mouse (*JAK2*^V617F^ /*THPO*^del/+^) delayed the erythrocytosis until 15 weeks, completely restored the neutrophil leukocytosis and thrombocytosis (Fig 1C), spleen weight (Fig 4) and marrow and spleen histology (Fig 5). Plasma THPO was reduced to below the level of the *THPO*^del/+^ mouse, indicating increased THPO utilization in the presence of *JAK2*^V617F^ (Fig 6). Thus, despite the presence of MPL with a constitutively-active JAK2, THPO-mediated signaling through MPL was still required for full expression of the PV phenotype in this *JAK2*^V617F^ transgenic mouse model.

### The marrow CD150+CD48- HSC compartment was expanded in the *JAK2*^V617F^ transgenic mouse and reduced in the absence of the *MPL* or *THPO* genes

**Fig 7:**
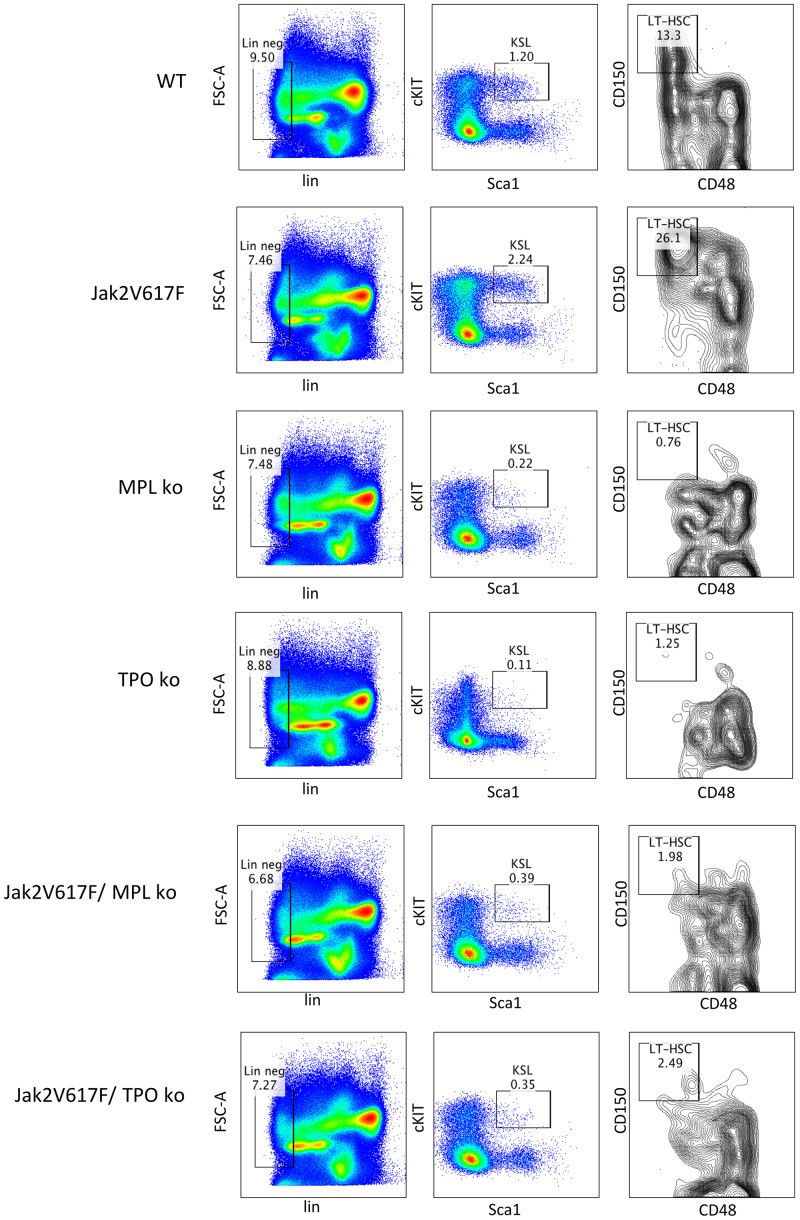
*MPL*^del^ and *THPO*^del^ genotypes reduce the LT-HSC (CD150+CD48-) population in a *JAK2*^V617F^ transgenic mouse model of PV. Flow cytometry of marrow LT-HSC at > 16 weeks in wild-type (n = 4), *MPL*^del/del^ (n = 3) and *THPO*^del/del^ (n = 3) mice and *JAK2*^V617^ (n = 4), *JAK2*^V617F^*/MPL*^del/del^ (n = 3) and *JAK2*^V617F^*/THPO*^del/del^ (n = 3) transgenic mice. The marrow LT-HSC population was 13% of the total LSK population in wild-type mice, 26% of the total LSK population in *JAK2*^V617F^ transgenic mice and 0.76% and 1.25% respectively in the *MPL*^del/del^ and *THPO*^del/del^ mice and 1.98 and 2.08% respectively of the total LSK population in *JAK2*^V617F^*/MPL*^del/del^ and *JAK2*^V617F^*/THPO*^del/del^ transgenic mice. WT vs *JAK2*^V617F^, P <0.029; *MPL*^del/del^ vs *JAK2*^V617F^, P < 0.022; *THPO*^del/del^ vs *JAK2*^V617F^, P <0.021; *JAK2*^V617F^ vs *JAK2*^V617F^*/MPL*^del/del^, P < 0.023; *JAK2*^V617F^ vs *JAK2*^V617F^*/THPO*^del/del^, P < 0.034. The number (n) of mice of each genotype studied is in parentheses.

MPL-THPO signaling is critical for HSC function and survival [48], therefore, we examined by flow cytometry the effect of loss of *MPL* or *THPO* on the number of marrow LT-HSC (CD150+CD48-) in the *JAK*2^V617F^ transgenic mouse. As shown in Fig 7, after week 16, the LT-HSC population was expanded 2 fold in the *JAK2*^V617F^ transgenic mouse compared to the wild-type mouse, but in the absence of either the *MPL* or *THPO* gene, the LT-HSC population in *JAK2*^V617F^ transgenic mice was reduced to the level of *MPL*^del/del^ or *THPO*^del/del^ mice. These results demonstrate that marrow LT-HSC in the *JAK2*^V617F^ transgenic mouse were dependent on THPO for marrow function.

## Discussion

The discovery that MPL protein expression was impaired in *JAK2*^V617F^-positive PV, PMF [39] and ET [40] was counterintuitive because MPL is the only hematopoietic growth factor receptor in HSC and the MPN are characterized by apparently autonomous myeloproliferation [49]. Moreover, impaired MPL protein expression appeared to be universal in the MPN since it was also associated with *MPL* [38,50] and *CALR* [29] mutations as well as with germline *MPL* SNP in the MPL distal CRHD causing familial thrombocytosis [32,33].

The potential mechanisms for impaired MPL expression include *MPL* mutations, increased MPL turnover, or incomplete post-translational processing. CAMT is due to *MPL* mutations [21], usually in the distal CRHD, while all three mechanisms are responsible for impaired MPN MPL expression [2,27,41,51]; germline SNP causing impaired MPL expression, which are also located in the distal CRHD, appear to involve impaired post-translational processing [51,52].

MPL is produced as an incompletely-glycosylated 80 kDa protein, which is fully glycosylated in the Golgi to a 95 kDa mature protein with JAK2 as its obligate chaperone [12]. Normally, both immature and mature MPL proteins are expressed at the cell-surface and both are THPO-responsive [41,53]. All *MPL* SNP or MPN driver mutations, however, result in impaired terminal MPL glycosylation in the distal CRHD; JAK2^V617F^ imposes an additional defect. JAK2 is responsible for enhancing MPL stability and recycling [12,41] but JAK2^V617F^ increases MPL ubiquitination and proteasomal degradation, resulting in decreased MPL recycling and half-life, predominantly involving mature MPL [41].

Importantly, impaired MPL expression does not affect megakaryocyte maturation or platelet production [15,16] but impairs plasma THPO clearance by these cells [54], increasing the stimulus for HPC proliferation, either unrestricted (*MPL*^S505N^, *MPL*^*W515* K/L^, *CALR*^*del/+*^ and *JAK2*^V617F^), or restricted to megakaryopoiesis (*MPL*^K39N^ and *MPL*^P106L^) because sufficient cell-surface MPL is still expressed in HCP for this purpose [53]. Indeed, the MPN phenotype partly mirrors that of mice [24,25] or humans with constitutive THPO production [36], a phenotype reversible in the mice by abrogation of THPO production [25].

Since MPL is essential to maintain HSC quiescence and survival in the marrow osteoblastic niche [17,18,55], while it is also responsible for THPO catabolism [54], we postulated that impaired MPL expression was central to MPN phenotypic behavior, causing myeloproliferation by HPC and eventually myelofibrosis due to increased circulating THPO, depending on the MPN driver mutation allele burden, while paradoxically permitting loss of HSC from marrow.

To test this hypothesis, we chose a *JAK2*^V617F^ transgenic mouse model that recapitulated the natural history of PV [45]. Not surprisingly, when bred to an *MPL*^del/del^ mouse, there was abrogation of the PV phenotype and a reduction in marrow HSC, which could be partially restored with expression of one *MPL* allele. This confirms a central role for *MPL* in this transgenic mouse model of PV, and indicates that the lower plasma THPO level compared to the wild-type mouse was due to increased THPO consumption by the JAK2^V617F^-mediated expansion of the megakaryocyte and circulating platelet pools, a feature also seen with *MPL* and *CALR* mutations [56].

To examine the role of THPO on the *JAK2*^V617F^ transgenic mouse phenotype, we bred this mouse with a *THPO*^del/del^ mouse. Surprisingly, there was modification of the PV phenotype including reversal of the osteomyelofibrosis and reduction in marrow HSC despite the fact there was biallelic expression of a functional MPL with *JAK2*^V617F^ as its tyrosine kinase. Restoration of a single *THPO* allele was sufficient to restore the PV phenotype, in contrast to incomplete restoration with a single *MPL* (*JAK*2^V617^/*MPL*^*del/+*^). This observation indicates that constitutive MPL signaling alone through JAK2^V617F^ was insufficient to support the full PV phenotype in this transgenic mouse model.

Our observations of abrogation of the PV phenotype by THPO gene deletion appear at odds with the results of the study of Sangkhae et al [57]. That study, however, employed a different *JAK2*^V617F^ transgenic mouse model with an ET phenotype [35] and only 16 weeks of observation, rendering their results not comparable to our *JAK2*^V617F^ transgenic mouse model, which recapitulated the natural history of PV, but required over 33 weeks of observation for full expression of the disease phenotype. Furthermore, Sangkhae et al claimed that THPO was not necessary for expression of the ET phenotype in their mouse model. However, In agreement with our observations, thrombocytosis was abrogated and in vitro HPC proliferation, megakaryocyte number and size, and spleen size were reduced in their *JAK2*^V617F^/*THPO*^del/del^ mice, indicating THPO dependence in their *JAK2*^V617F^ transgenic mouse model.

An important consideration is whether observations in a *JAK2*^V617F^ transgenic mouse model of PV with normal mouse MPL expression, can be extrapolated to human PV. In the wild-type mouse, the average platelet count is ~1,000x10^9^/L and the plasma THPO level is ~400 pg/mL, while in humans, the average platelet count is ~250 x10^9^/L and the plasma THPO level is ~55 pg/m [58]. Furthermore, mice, unlike humans, can survive without expressing MPL [46]. These differences, however, may be deceiving.

Humans with the benign germline SNP, K39N (*MPL* Baltimore [32]), however, actually recapitulate mouse hematopoiesis with thrombocytosis, an elevated plasma THPO level and impaired MPL cell-surface expression. Conversely, when the asparagine at residue 39 in wild-type mice is removed, their platelet counts fall into the human normal range as do their plasma THPO levels [59], indicating that mouse hematopoiesis can also in part recapitulate human hematopoiesis.

Similarly, THPO metabolism in the *JAK2*^V617F^ transgenic mouse model of PV also represents a difference between this model and the human disease since plasma THPO is elevated in human PV. It does, however, emphasize that the MPN are hematopoietic growth factor-dependent disorders, particularly in the *JAK2*^V671F^ heterozygous state. For example, in human PV, BFU-E heterozygous for *JAK2*^V617F^ were responsive in vitro to erythropoietin in a manner similar to normal BFU-E [60] and HPC hematopoietic growth factor-responsiveness was also observed in vitro with *MPL* [38] and *CALR* [4,31] mutations. In this regard, our in vitro HPC colony-forming assays indicate that JAK2^V617F^ expression alone accounted for approximately 50% of marrow GFU-GM colony formation (Fig 2A) and 30% of CFU-Mk colonies (Fig 2D), with the rest dependent on THPO stimulation.

The reduction in marrow LT HSC in the absence of MPL or THPO in our studies also supports the contention that the MPN are hematopoietic growth factor-dependent-diseases. Importantly, an MPL small molecule antagonist preferentially inhibited *JAK2*^V617F^-positive PV HSC proliferation both in vitro and in vivo compared to normal HSC [61], while in vitro, PV HPC^68^ and murine cell lines expressing activating *MPL* [38] or *CALR* [4,31] mutations were still THPO-responsive despite the presence of constitutively-activated JAK2. Additionally, in vivo exposure of wild-type mice to an MPL antagonist antibody permitted nonmyeloablative bone marrow transplantation, substantiating the need for THPO to maintain LT-HSC in their marrow niches [18]. Importantly, while MPL absence leads to CAMT in humans, MPL expression is impaired but not absent in MPN HSC and HPC and as demonstrated experimentally [61], should give normal HSC and HCP a survival advantage in the presence of a THPO antagonist. Finally, the development of biologically effective in vivo silencers of THPO production [62, 63] makes testing this therapeutic approach feasible.

**Fig 8:**
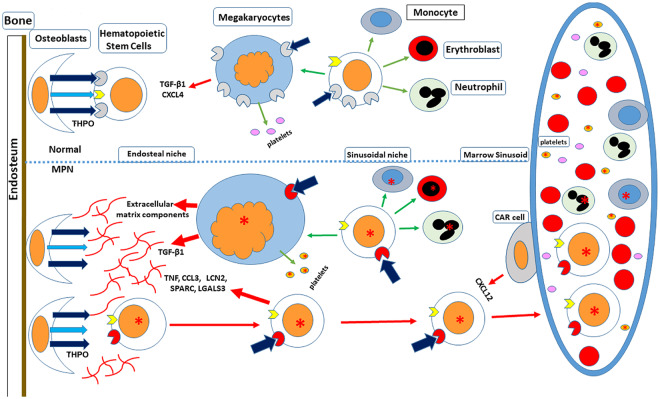
A schematic representation of the effect of impaired MPL expression on the behavior of bone marrow HSC. In the endosteal niche, HSCs are tethered to osteoblasts by a variety of adhesive proteins (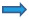) and their receptors (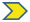), and thrombopoietin (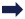) and its receptor, MPL, (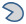), and are maintained in a quiescent state by platelet factor 4 (CXCL4) and TGF-β, secreted by closely opposed megakaryocytes. In the MPN, reduced cell surface expression of MPL (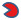) facilitates egress of hematopoietic stem cells expressing a driver mutation (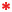) from the endosteal niche followed by migration to the sinusoidal niche, expedited by the stromal cell-derived growth factor-1 (CXCL12), secreted by CXCL12-abundant reticular (CAR) cells; there the stem cells can either differentiate (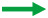) in response to THPO, the plasma level of which is high in the MPN due to reduced platelet MPL cell surface expression, or leave the marrow (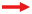) via the sinusoids for residence in the spleen or other organs. MPN HSCs, like chronic myeloid leukemia HSC, also produce inflammatory cytokines, including tumor necrosis factor (TNF), macrophage inflammatory protein 1α (CCL3), osteonectin (SPARC) and Lipocalin 2 (LCN2), which alter the marrow cellular microenvironment, suppressing normal hematopoietic stem cells and promoting myelofibrosis (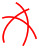).

From our observations, therefore, we postulate that impaired MPL expression in the MPN results in an inappropriately high plasma THPO level through failure of THPO clearance by MPN platelets and megakaryocytes, which augments activated JAK2 signaling in HPC, while weakening the ability of HSC to remain in the marrow osteoblastic niche (Fig 8). With time, marrow HSC loss due to differentiation or migration and sequestration in the spleen, and continued megakaryocyte stimulation by the elevated plasma THPO, could produce a PMF phenotype, regardless of the MPN driver mutation. From this perspective, the MPN are in part hematopoietic growth factor-dependent disorders and targeting the MPL-THPO axis could be an effective, nonmyelotoxic therapeutic strategy.

## Supporting information

S1 Fig Breeding strategy to obtain *JAK2*^V617F^ transgenic mice in the desired background. *JAK2*^V617F^ transgenic mice were crossed into the *MPL* knockout background as shown with the ratios of each genotype obtained over the total number of progeny from the matings. Identical breeding schemes were used to obtain the *JAK2*^V617F^/*THPO*^del/del^ and *JAK2*^V617F^/*THPO*^del/+^ transgenic mouse genotypes.
(DOCX)

S1 Table Primers used for genotyping the *JAK2*^V617F^, *MPL*^del/del^ and *THPO*^del/del^ mice. (DOCX)

S2 Table Blood counts of the 10 mouse genotypes. (DOCX)

S3 Table Statistical analysis of the blood counts from the 10 mouse genotypes. (DOCX)

## References

[1] James C, Ugo V, Le Couedic JP, Staerk J, Delhommeau F, Lacout C, et al A unique clonal JAK2 mutation leading to constitutive signalling causes polycythaemia vera. Nature 2005;434:1144–1148. 15793561 10.1038/nature03546

[2] Pikman Y, Lee BH, Mercher T, McDowell E, Ebert BL, Gozo M, et al MPLW515L Is a Novel Somatic Activating Mutation in Myelofibrosis with Myeloid Metaplasia. PLoS.Med. 2006;3:e270. 16834459 10.1371/journal.pmed.0030270 PMC1502153

[3] Nangalia J, Massie CE, Baxter EJ, Nice FL, Gundem G, Wedge DC, et al Somatic CALR mutations in myeloproliferative neoplasms with nonmutated JAK2. N.Engl.J.Med. 2013;369:2391–2405. 24325359 10.1056/NEJMoa1312542 PMC3966280

[4] Klampfl T, Gisslinger H, Harutyunyan AS, Nivarthi H, Rumi E, Milosevic JD, et al Somatic mutations of calreticulin in myeloproliferative neoplasms. N Engl J Med 2013;369:2379–2390. 24325356 10.1056/NEJMoa1311347

[5] Vainchenker W, Dusa A, Constantinescu SN. JAKs in pathology: role of Janus kinases in hematopoietic malignancies and immunodeficiencies. Semin.Cell Dev.Biol. 2008;19:385–393. 18682296 10.1016/j.semcdb.2008.07.002

[6] Shimoda K, Feng J, Murakami H, Nagata S, Watling D, Rogers NC, et al Jak1 plays an essential role for receptor phosphorylation and Stat activation in response to granulocyte colony-stimulating factor. Blood 1997;90:597–604. 9226159

[7] Spivak JL, Pham T, Isaacs M, Hankins WD. Erythropoietin is both a mitogen and a survival factor. Blood 1991;77:1228–1233. 1705834

[8] Koury MJ, Bondurant MC. Erythropoietin retards DNA breakdown and prevents programmed death in erythroid progenitor cells. Science 1990;248:378–381. 2326648 10.1126/science.2326648

[9] Verstovsek S, Mesa RA, Gotlib J, Levy RS, Gupta V, DiPersio JF, et al A double-blind, placebo-controlled trial of ruxolitinib for myelofibrosis. N.Engl.J.Med. 2012;366:799–807. 22375971 10.1056/NEJMoa1110557 PMC4822164

[10] Mullally A, Lane SW, Ball B, Megerdichian C, Okabe R, Al Shahrour F, et al Physiological Jak2V617F expression causes a lethal myeloproliferative neoplasm with differential effects on hematopoietic stem and progenitor cells. Cancer Cell 2010;17:584–596. 20541703 10.1016/j.ccr.2010.05.015 PMC2909585

[11] Wang X, Ye F, Tripodi J, Hu CS, Qui J, Najfeld V, et al JAK2 inhibitors do not affect stem cells present in the spleens of patients with myelofibrosis. Blood 2014;124:2987–2995. 25193869 10.1182/blood-2014-02-558015 PMC4224194

[12] Royer Y, Staerk J, Costuleanu M, Courtoy PJ, Constantinescu SN. Janus kinases affect thrombopoietin receptor cell surface localization and stability. J.Biol.Chem. 2005;280:27251–27261. 15899890 10.1074/jbc.M501376200

[13] Borge OJ, Ramsfjell V, Veiby OP, Murphy MJ, Lok S, Jacobsen SE, et al Thrombopoietin, but not erythropoietin promotes viability and inhibits apoptosis of multipotent murine hematopoietic progenitor cells in vitro. Blood 1996;88:2859–2870. 8874182

[14] Goncalves F, Lacout C, Villeval JL, Wendling F, Vainchenker W, Dumenil D. Thrombopoietin does not induce lineage-restricted commitment of Mpl-R expressing pluripotent progenitors but permits their complete erythroid and megakaryocytic differentiation. Blood 1997;89:3544–3553. 9160659

[15] de Sauvage FJ, Carver-Moore K, Luoh SM, Ryan A, Dowd M, Eaton DL. Physiological regulation of early and late stages of megakaryocytopoiesis by thrombopoietin. J Exp.Med 1996;183:651–656. 8627177 10.1084/jem.183.2.651 PMC2192470

[16] Levin J, Cocault L, Demerens C, Challier C, Pauchard M, Jensen et al Thrombocytopenic c-mpl(-/-) mice can produce a normal level of platelets after administration of 5-fluorouracil: the effect of age on the response. Blood 2001;98:1019–1027. 11493447 10.1182/blood.v98.4.1019

[17] Qian H, Buza-Vidas N, Hyland CD, Jensen CT, Antonchuk J, Mansson R, et al Critical role of thrombopoietin in maintaining adult quiescent hematopoietic stem cells. Cell Stem Cell 2007;1:671–684. 18371408 10.1016/j.stem.2007.10.008

[18] Yoshihara H, Arai F, Hosokawa K, Hagiwara T, Takubo K, Nakamura Y, et al Thrombopoietin/MPL signaling regulates hematopoietic stem cell quiescence and interaction with the osteoblastic niche. Cell Stem Cell 2007;1:685–697. 18371409 10.1016/j.stem.2007.10.020

[19] Murone M, Carpenter DA, de Sauvage FJ. Hematopoietic deficiencies in c-mpl and TPO knockout mice. Stem Cells 1998;16:1–6. 10.1002/stem.160001 9474742

[20] Ballmaier M, Germeshausen M. Advances in the understanding of congenital amegakaryocytic thrombocytopenia. Br J Haematol. 2009;146:3–16. 19388932 10.1111/j.1365-2141.2009.07706.x

[21] Geddis AE. Congenital amegakaryocytic thrombocytopenia. Pediatr Blood Cancer 2011;57:199–203. 21337678 10.1002/pbc.22927

[22] Wendling F, Varlet P, Charon M, Tambourin P. MPLV: a retrovirus complex inducing an acute myeloproliferative leukemic disorder in adult mice. Virology 1986;149:242–246. 3004028 10.1016/0042-6822(86)90125-x

[23] Souyri M, Vigon I, Penciolelli JF, Heard JM, Tambourin P, Wendling F. A putative truncated cytokine receptor gene transduced by the myeloproliferative leukemia virus immortalizes hematopoietic progenitors. Cell 1990;63:1137–1147. 2175677 10.1016/0092-8674(90)90410-g

[24] Villeval JL, Cohen-Solal K, Tulliez M, Giraudier S, Guichard J, Burstein SA, et al High thrombopoietin production by hematopoietic cells induces a fatal myeloproliferative syndrome in mice. Blood 1997;90:4369–4383. 9373248

[25] Yan XQ, Lacey D, Hill D, Chen Y, Fletcher F, Hawley RG, et al A model of myelofibrosis and osteosclerosis in mice induced by overexpressing thrombopoietin (mpl ligand): reversal of disease by bone marrow transplantation. Blood 1996;88:402–409. 8695786

[26] Villeval JL, Metcalf D, Johnson GR. Fatal polycythemia induced in mice by dysregulated erythropoietin production by hematopoietic cells. Leukemia 1992;6:107–115. 1552741

[27] Ding J, Komatsu H, Wakita A, Kato-Uranishi M, Ito M, Satoh A, et al Familial essential thrombocythemia associated with a dominant-positive activating mutation of the c-MPL gene, which encodes for the receptor for thrombopoietin. Blood 2004;103:4198–4200. 14764528 10.1182/blood-2003-10-3471

[28] Williams DM, Kim AH, Rogers O, Spivak JL, Moliterno AR. Phenotypic variations and new mutations in JAK2 V617F-negative polycythemia vera, erythrocytosis, and idiopathic myelofibrosis. Exp.Hematol. 2007;35:1641–1646. 17920755 10.1016/j.exphem.2007.08.010 PMC2149910

[29] Chachoua I, Pecquet C, El-Khoury M, Nivarthi H, Albu RI, Marty C, et al Thrombopoietin receptor activation by myeloproliferative neoplasm associated calreticulin mutants. Blood 2016;127:1325–1335. 26668133 10.1182/blood-2015-11-681932

[30] Elf S, Abdelfattah NS, Baral AJ, Beeson D, Rivera JF, Ko A, et al Defining the requirements for the pathogenic interaction between mutant calreticulin and MPL in MPN. Blood 2018;131:782–786. 29288169 10.1182/blood-2017-08-800896 PMC5814933

[31] Araki M, Yang Y, Masubuchi N, Hironaka V, Takei H, Morishita S, et al Activation of the thrombopoietin receptor by mutant calreticulin in CALR-mutant myeloproliferative neoplasms. Blood 2016;127:1307–1316. 26817954 10.1182/blood-2015-09-671172

[32] Moliterno AR, Williams DM, Gutierrez-Alamillo LI, Salvatori R, Ingersoll RG, Spivak JL. Mpl Baltimore: a thrombopoietin receptor polymorphism associated with thrombocytosis. Proc Natl.Acad Sci U.S.A 2004;101:11444–11447. 15269348 10.1073/pnas.0404241101 PMC509220

[33] El Harith eL, Roesl C, Ballmaier M, Germeshausen M, Frye-Boukhriss H, von Neuhoff N, et al Familial thrombocytosis caused by the novel germ-line mutation p.Pro106Leu in the MPL gene. Br.J.Haematol. 2009;144:185–194. 19036112 10.1111/j.1365-2141.2008.07430.x

[34] Lannutti BJ, Epp A, Roy J, Chen J, Josephson NC. Incomplete restoration of Mpl expression in the mpl-/- mouse produces partial correction of the stem cell-repopulating defect and paradoxical thrombocytosis. Blood 2009;113:1778–1785. 18796624 10.1182/blood-2007-11-124859 PMC2647669

[35] Tiedt R, Coers J, Ziegler S, Wiestner A, Hao-Shen H, Bor, et al Pronounced thrombocytosis in transgenic mice expressing reduced levels of Mpl in platelets and terminally differentiated megakaryocytes. Blood 2009;113:1768–1777. 18845793 10.1182/blood-2008-03-146084

[36] Wiestner A, Schlemper RJ, van der Maas AP, Skoda RC. An activating splice donor mutation in the thrombopoietin gene causes hereditary thrombocythaemia. Nat.Genet. 1998;18:49–52. 9425899 10.1038/ng0198-49

[37] Posthuma HL, Skoda RC, Jacob FA, van der Maas AP, Valk PJ, Posthuma EF. Hereditary thrombocytosis not as innocent as thought? Development into acute leukemia and myelofibrosis. Blood 2010;116:3375–3376. 21030572 10.1182/blood-2010-06-290718

[38] Ding J, Komatsu H, Iida S, Yano H, Inagaki A, Mori F, et al The Asn505 mutation of the c-MPL gene, which causes familial essential thrombocythemia, induces autonomous homodimerization of the c-Mpl protein due to strong amino acid polarity. Blood 2009;114:3325–3328. 19483125 10.1182/blood-2008-04-149047

[39] Moliterno AR, Hankins WD, Spivak JL. Impaired expression of the thrombopoietin receptor by platelets from patients with polycythemia vera. N.Engl.J.Med. 1998;338:572–580. 9475764 10.1056/NEJM199802263380903

[40] Horikawa Y, Matsumura I, Hashimoto K, Shiraga M, Kosugi S, Tadokoro S, et al Markedly reduced expression of platelet c-mpl receptor in essential thrombocythemia. Blood 1997;90:4031–4038. 9354672

[41] Pecquet C, Diaconu CC, Staerk J, Girardot M, Marty C, Royer Y, et al Thrombopoietin receptor down-modulation by JAK2 V617F: restoration of receptor levels by inhibitors of pathologic JAK2 signaling and of proteasomes. Blood 2012;119:4625–4635. 22378845 10.1182/blood-2011-08-372524

[42] Li J, Xia Y, Kuter DJ. The platelet thrombopoietin receptor number and function are markedly decreased in patients with essential thrombocythaemia. Br.J Haematol. 2000;111:943–953. 11122159

[43] Wang JC, Chen C, Novetsky AD, Lichter SM, Ahmed F, Friedberg NM. Blood thrombopoietin levels in clonal thrombocytosis and reactive thrombocytosis. Am.J.Med. 1998;104:451–455. 9626028 10.1016/s0002-9343(98)00090-4

[44] Wang JC, Chen C, Lou LH, Mora M. Blood thrombopoietin, IL-6 and IL-11 levels in patients with agnogenic myeloid metaplasia. Leukemia 1997;11:1827–1832. 9369414 10.1038/sj.leu.2400846

[45] Xing S, Wanting TH, Zhao W, Ma J, Wang S, Xu X, et al Transgenic expression of JAK2V617F causes myeloproliferative disorders in mice. Blood 2008;111:5109–5117. 18334677 10.1182/blood-2007-05-091579 PMC2384138

[46] Gurney AL, Carver-Moore K, de Sauvage FJ, Moore MW. Thrombocytopenia in c-mpl-deficient mice. Science 1994;265:1445–1447. 8073287 10.1126/science.8073287

[47] Pietras EM, Reynaud D, Kang YA, Carlin D, Calero-Nieto FJ, Leavitt AD, et al Functionally Distinct Subsets of Lineage-Biased Multipotent Progenitors Control Blood Production in Normal and Regenerative Conditions. Cell Stem Cell 2015;17:35–46. 26095048 10.1016/j.stem.2015.05.003 PMC4542150

[48] Gurney AL, de Sauvage FJ. Dissection of c-Mpl and thrombopoietin function: studies of knockout mice and receptor signal transduction. Stem Cells 1996;14 Suppl 1:116–123. 11012211 10.1002/stem.5530140715

[49] Prchal JF, Axelrad AA. Letter: Bone-marrow responses in polycythemia vera. N.Engl.J.Med. 1974;290:1382. 10.1056/nejm197406132902419 4827655

[50] Marty C, Chaligne R, Lacout C, Constantinescu SN, Vainchenker W, Villeval JL. Ligand-independent thrombopoietin mutant receptor requires cell surface localization for endogenous activity. J.Biol.Chem. 2009;284:11781–11791. 19261614 10.1074/jbc.M808703200 PMC2673247

[51] Moliterno AR, Spivak JL. Posttranslational processing of the thrombopoietin receptor is impaired in polycythemia vera. Blood 1999;94:2555–2561. 10515857

[52] Favale F, Messaoudi K, Varghese LN, Boukour S, Pecquet C, Gryshkova V, et al An incomplete trafficking defect to the cell-surface leads to paradoxical thrombocytosis for human and murine MPL P106L. Blood 2016;128:3146–3158. 28034873 10.1182/blood-2016-06-722058

[53] Cleyrat C, Darehshouri A, Steinkamp MP, Vilaine M, Boassa D, Ellisman MH, et al Mpl traffics to the cell surface through conventional and unconventional routes. Traffic. 2014;15:961–982. 24931576 10.1111/tra.12185 PMC4141020

[54] Kuter DJ, Rosenberg RD. The reciprocal relationship of thrombopoietin (c-Mpl ligand) to changes in the platelet mass during busulfan-induced thrombocytopenia in the rabbit. Blood 1995;85:2720–2730. 7742532

[55] Kohlscheen S, Wintterle S, Schwarzer A, Kamp C, Brugman MH, Breuer DC, et al Inhibition of Thrombopoietin/Mpl Signaling in Adult Hematopoiesis Identifies New Candidates for Hematopoietic Stem Cell Maintenance. PLoS.ONE. 2015;10:e0131866. 26147434 10.1371/journal.pone.0131866 PMC4493002

[56] Li J, Prins D, Park HJ, Grinfeld J, Gonzalez-Arias C, Loughran S, et al Mutant calreticulin knockin mice develop thrombocytosis and myelofibrosis without a stem cell self-renewal advantage. Blood 2018;131:649–661. 29282219 10.1182/blood-2017-09-806356

[57] Sangkhae V, Etheridge SL, Kaushansky K, Hitchcock IS. The thrombopoietin receptor, MPL, is critical for development of a JAK2V617F-induced myeloproliferative neoplasm. Blood 2014;124:3956–3963. 25339357 10.1182/blood-2014-07-587238 PMC4271181

[58] Seiki Y, Sasaki Y, Hosokawa K, Saito C, Sugimori N, Yamazaki H, et al Increased plasma thrombopoietin levels in patients with myelodysplastic syndrome: a reliable marker for a benign subset of bone marrow failure. Haematologica 2013;98:901–907. 23403320 10.3324/haematol.2012.066217 PMC3669446

[59] Spivak J, Williams D, Zhao Z, Rogers O, Duffield A, Hankins WD, et al MPL SV, a Unique MPN MPL Splice Variant, Provokes a Fulminant Myeloid Leukemia in a *JAK2* V617F Transgenic Mouse Model of Polycythemia Vera [abstract]. Blood 2015;126:484.

[60] Dupont S, Masse A, James C, Teyssandier I, Lecluse Y, Larbret F, et al The JAK2 617V>F mutation triggers erythropoietin hypersensitivity and terminal erythroid amplification in primary cells from patients with polycythemia vera. Blood 2007;110:1013–1021. 17389763 10.1182/blood-2006-10-054940

[61] Wang X, Haylock D, Hu CS, Kowalczyk W, Jiang T, Qui J, et al A thrombopoietin receptor antagonist is capable of depleting myelofibrosis hematopoietic stem and progenitor cells. Blood 2016;127:3398–3409. 27114459 10.1182/blood-2015-10-674465 PMC4929928

[62] Shirai T, Revenko AS, Tibbitts J, Ngo ATP, Mitrugno A, Healy L D, et al Hepatic thrombopoietin gene silencing platelet count and breast cancer progression in transgenic MMTV-PyMT mice. Blood Adv 2019;3: 3080–3091. 31648335 10.1182/bloodadvances.2019000250 PMC6849943

[63] Desai D, Borodovsky A, Davis WP, Degaonkar R, Yucius K, D’Angelo K, et al Development of Liver-Specific Thrombopoietin Targeted Sirnas: Impact on Platelet Count, Megakaryocyte Mass, and Hematopoietic Progenitors in Normal and MPN Murine Models [abstract]. Blood 2018;132: 4329.

