# Supplementary material for: Thrombopoietin is required for full phenotype expression in a JAK2V617F transgenic mouse model of polycythemia vera
Source: PLoS One. 2020 Jun 1;15(6):e0232801. doi: 10.1371/journal.pone.0232801 (PMC7263591; doi:10.1371/journal.pone.0232801)
Supplement: S1 Fig — JAK2V617F transgenic mice were crossed into the MPL knockout background as shown with the ratios of each genotype obtained over the total number of progeny from the matings. Identical breeding schemes were used to obtain the JAK2V617F/THPOdel/del and JAK2V617F/THPOdel/+ transgenic mouse genotypes. (DOCX) [file pone.0232801.s001.docx]

**S1 Figure Legend**

**Breeding strategy to obtain *JAK2*^V617F^ transgenic mice in the desired background.**

*JAK2*^V617F^ transgenic mice were crossed into the *MPL* knockout background as shown with the expected ratios of the progeny of each genotype mating shown with the ratios of the progeny actually obtained from the matings. An identical breeding scheme was used to obtain the *JAK2*^V617F^/*THPO*^del/del^ and *JAK2*^V617F^/*THPO*^del/+^ transgenic mouse genotypes.

**
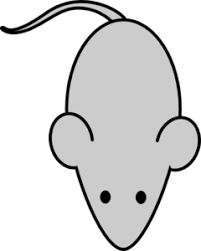

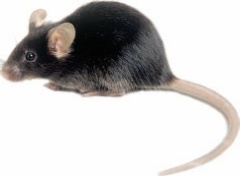
S1 Figure**

**X**

*MPL*^del/del^ mouse

*JAK2*^V617F^ transgenic mouse

**
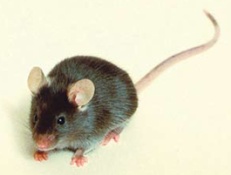
**


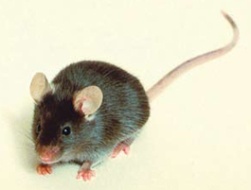

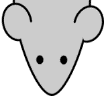


**50%** *JAK2*^V617F^/*MPL*^del/+^

**50%** *MPL*^del/+^

29/50

21/50


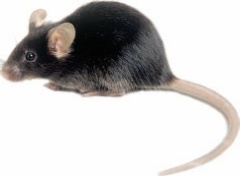


*MPL*^del/del^

*JAK2*^V617F^/*MPL*^del/+^


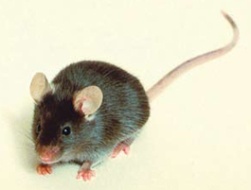

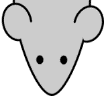


**X**

**
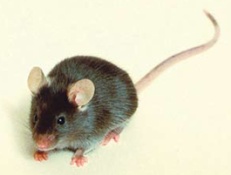

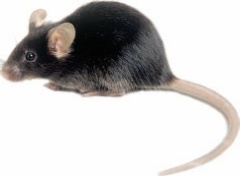
**


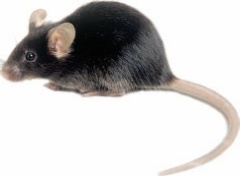

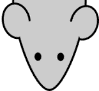

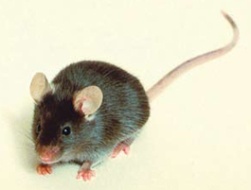

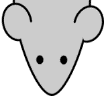


**25%** *MPL*^del/del^

**25%** *MPL*^del/+^

**25%** *JAK2*^V617F^/*MPL*^del/+^

**25%** *JAK2*^V617F^/*MPL*^del/del^

18/119 27/119 39/119 35/119
