# Supplementary material for: Thrombopoietin is required for full phenotype expression in a JAK2V617F transgenic mouse model of polycythemia vera
Source: PLoS One. 2020 Jun 1;15(6):e0232801. doi: 10.1371/journal.pone.0232801 (PMC7263591; doi:10.1371/journal.pone.0232801)
Supplement: S1 Table — (DOCX) [file pone.0232801.s002.docx]

| **S1 Table. Primers used for genotyping the *JAK2*^V617F^, *MPL*^del/del^ and *THPO*^del/del^ mice.** | | | |
| --- | --- | --- | --- |
| Genotype | Primers | | Reference |
| *JAK2^V^*^617F^ transgenic mouse | End Point PCR | | Blood. 2008; 111:5109-5117. |
|  | *JAK2*^V617F^ sense: | 5'- TACAACCTCAGTGGGACAAAGAAGAAC -3' |  |
|  | *JAK2*^V617F^ antisense: | 5'- CCATGCCAACTGTTTAGCAACTTCA -3' |  |
|  | | | |
| *MPL*^del/del^ mouse | End Point PCR | | Science. 1994; 265:1445-1447. |
|  | *MPL* wt sense: | 5'- TCGATCTAGAGCCCCGTGCATGCCCCCTGTATT -3' |  |
|  | *MPL* wt antisense: | 5'- TCGAATCGATACCCACATCGTCCTGAAAGACTA -3' |  |
|  | *MPL* Neo sense: | 5'- CAAGACCGACCTGTCCGGTG -3' |  |
|  | *MPL* Neo antisense: | 5'- AGGCGATAGAAGGCGATGCG -3' |  |
|  |  |  |  |
| *THPO*^del/del^ mouse | End Point PCR | | J. Exp. Med. 1966; 183:651-656. |
|  | p.*TPHO*.SalI | 5'- GTCGACCCTTTGTCTATCCCT -3' |  |
|  | p.*THPO*.SacI | 5'- GGTGAATGTAACCTGGGATAA -3' |  |
|  | *THPO* Neo sense: | 5'- CGGTTCTTTTTGTCAAGAC -3' |  |
|  | *THPO* Neo antisense: | 5'- ATCCTCGCCGTCGGGCATGC -3' |  |
