# Supplementary material for: Thrombopoietin is required for full phenotype expression in a JAK2V617F transgenic mouse model of polycythemia vera
Source: PLoS One. 2020 Jun 1;15(6):e0232801. doi: 10.1371/journal.pone.0232801 (PMC7263591; doi:10.1371/journal.pone.0232801)
Supplement: S2 Table — (DOCX) [file pone.0232801.s003.docx]

**S2 Table. Blood counts of the 11 mouse genotypes**
